# Visual representation of rational belief revision: another look at the Sleeping Beauty problem

**DOI:** 10.3389/fpsyg.2014.01232

**Published:** 2014-10-29

**Authors:** David R. Mandel

**Affiliations:** Socio-Cognitive Systems Section, Defence Research and Development Canada, Toronto Research Centre, Department of Psychology, York UniversityToronto, ON, Canada

**Keywords:** Bayesian reasoning, belief revision, visual representation, rationality, Sleeping Beauty problem

The coherence of probability judgments is influenced in predictable ways by people's internal representations of problems, which may be altered by the manner in which propositions are stated or “framed” (Mandel, 2008). Likewise, several studies find that probabilistic reasoning and judgment can be improved by externally representing statistical information visually (for a review, see Garcia-Retamero and Cokely, 2013). Visual representation is thought to facilitate performance by externalizing the set-subset relations among observational data. Although some studies have examined whether visual representations can improve Bayesian reasoning, they have tended to focus on the use of natural sampling trees (Sedlmeier and Gigerenzer, 2001), Euler circles (Sloman et al., 2003), or other means of representing set-subset relations.

However, visualization can aid reasoning and judgment even when problems do not involve natural or normalized frequency representations. Take the “Ann problem” adapted by Over (2007b):

Jack is looking at Ann but Ann is looking at George. Jack is a cheater but George is not. Is a cheater looking at a non-cheater?

(A) Yes (B) No (C) Cannot tell

In a variant of the problem, Toplak and Stanovich (2002) found that most people say they cannot tell, although the correct answer is yes. Wrong answers are common because most people do not consider the implications of the fact that Ann is either a cheater or she is not. As Over (2007b) notes, the logic of the excluded middle—namely, that all propositions of the form “*x* or not-*x*” are logically true—is often neglected.

Instead people seem to be guided by their sense of uncertainty about both of the dyadic relations in the problem, remaining unaware that their uncertainty should not preclude a more definite conclusion. As Over (2007a,b) suggests, logic trees, which represent possibilities on branches, can provide a useful visualization tool for overcoming such psychological barriers. If one were to draw out the two possibilities in the Ann problem—one in which cheater Jack looks at non-cheater Ann and the other in which cheater Ann looks at non-cheater George—the correct answer is evident. If you draw a logic tree showing the two possibilities (Ann as a cheater or as a non-cheater) and the “looking relations” that are entailed in each, it becomes evident that no matter what Ann is, a cheater will always look at a non-cheater. Who the cheater is and who the non-cheater is will differ depending on whether Ann is a cheater or not, but those details are irrelevant to the question. The logic tree also shows that it is impossible for a non-cheater to look at a cheater. However, in that case, one must attend to what is omitted from the set of possible worlds.

## The sleeping beauty problem

In the remainder of this paper, I explore the value of logic trees in representing alternative arguments by experts about normative belief updating. I focus on the Sleeping Beauty problem introduced by Elga (2000) and discussed shortly thereafter by Lewis (2001). My aim is twofold: First, I want to show how these authors' arguments may be represented and how the representations may be compared. Second, I want to propose a resolution of the disagreement over the problem that I believe is novel.

This is Lewis's description of the problem:

Researchers at Experimental Philosophy Laboratory have decided to carry out the following experiment. First they will tell Sleeping Beauty [SB] all that I am about to tell you in this paragraph, and they will see to it that she fully believes all she is told. Then on Sunday evening they will put her to sleep. On Monday they will awaken her briefly. At first they will not tell her what day it is, but later they will tell her that it is Monday. Then they will subject her to memory erasure. Perhaps they will again awaken her briefly on Tuesday. Whether they do will depend on the toss of a fair coin: if heads they will awaken her only on Monday, if tails they will awaken her on Tuesday as well. On Wednesday the experiment will be over and she will be allowed to wake up. The three possible brief awakenings during the experiment will be indistinguishable: she will have the same total evidence at her Monday awakening whatever the result of the coin toss may be, and if she is awakened on Tuesday the memory erasure on Monday will make sure that her total evidence at the Tuesday awakening is exactly the same as at the Monday awakening. However, she will be able, and she will be taught how, to distinguish her brief awakenings during the experiment from her Wednesday awakening after the experiment is over, and indeed from all other actual awakenings there have ever been, or ever will be.

Furthermore, assume that SB is a paragon of rationality and let us also assume for the sake of concreteness that the coin is tossed on Sunday night after SB is put to sleep. What subjective probability should she assign to heads (*H*) upon her awakening on Monday, and then again after she is told that it is Monday?

Elga and Lewis agree that SB will be in one of three states:

*H*_1_: Heads and it is Monday

*T*_1_: Tails and it is Monday

*T*_2_: Tails and it is Tuesday.

Elga starts out by imagining that SB knows that the coin lands on tails. Since *T*_1_ and *T*_2_ would be indistinguishable to SB, he argues that she should assign each the same probability: *P*(*T*_1_) = *P*(*T*_2_) = 1/2. Next, Elga imagines that SB knows it is Monday, arguing that SB should assign equal probability to *H*_1_ and *T*_1_ given the fact that the coin is fair. Thus, *P*(*H*_1_) = *P*(*T*_1_) = *P*(*T*_2_). Since these probabilities must sum to 1, each must equal 1/3. Therefore, Elga proposes that, on waking in an asynchronous state, SB should assign a 1/3 probability to heads, and that she should revise this probability to 1/2 after learning it is Monday.

Lewis disagrees. He starts out with the principle that the subjective probability of a future chance event should be equal to the known chances (Mellor, 1971; Lewis, 1980). Since the coin is fair, the known chances indicate *P*(*H*) = *P*(*T*) = 1/2. Lewis argues that on awakening SB has not learned anything new that would warrant belief revision. She has no new knowledge of her location. Like Elga, Lewis accepts that SB should regard *P*(*T*_1_) = *P*(*T*_2_). Given *P*(*T*) = *P*(*T*_1_ ∨ *T*_2_) = 1/2, and the disjunctive possibilities are equiprobable, *P*(*T*_1_) = *P*(*T*_2_) = 1/4.

Elga and Lewis agree that, upon learning it is Monday, SB should increase her subjective probability of heads by 1/6 after conditionalizing on the remaining possibilities. For Elga, *P*(*H* | *H*_1_ ∨ *T*_1_) = (1/3)/(2/3) = 1/2. For Lewis, *P*(*H* | *H*_1_ ∨ *T*_1_) = (1/2)/(3/4) = 2/3. Interestingly, Lewis does not apply his imaging rule for belief updating (Lewis, 1976) here, even though it arguably applies (Cozic, 2011; see also Baratgin, 2009).

**Figure 1:**
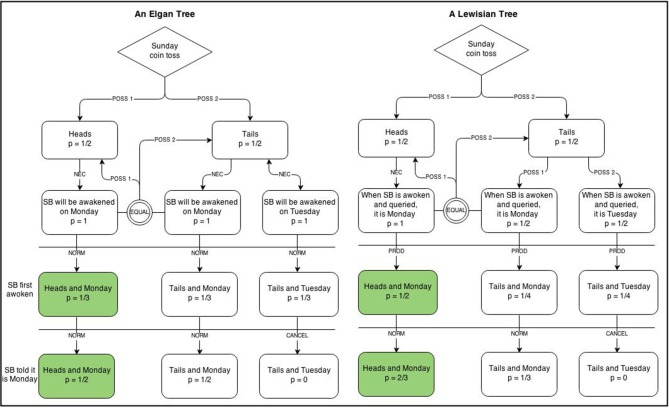
**Enhanced logic trees for Elga's (2000) and Lewis's (2001) positions on the Sleeping Beauty problem**. POSS, possibility; NEC, necessity; NORM, normalize; PROD, product.

The SB problem continues to prompt philosophical debate (e.g., Dorr, 2002; Horgan, 2004; Weintraub, 2004; Rosenthal, 2009; Baratgin and Walliser, 2010). In my own thinking about it, I have found it useful to externally visualize the alternative arguments using enhanced logic trees that also encode operations (e.g., normalization) or relation types (e.g., necessity). Figure 1 shows possible logic trees for Elga's “thirder” and Lewis's “halfer” positions. It reveals that the locus of disagreement is in the apportioning of probability to *T*_1_ and *T*_2_.

In Elga's analysis, these two centered possibilities each have a subjective probability of 1/2 since the coin toss outcome *T*, all agree, equals 1/2 and the Monday and Tuesday awakenings necessarily follow. Since *H*_1_ also equals 1/2, the probabilities must be normalized to constrain their sum to 1. This leads to each centered possibility having a probability of 1/3.

In Lewis's analysis, the same two centered possibilities, *T*_1_ and *T*_2_, each have a subjective probability of 1/4 because Lewis applies a principle of indifference to them. Given that the three centered possibilities are additive, normalization is not required and *H*_1_ remains 1/2.

The visualizations reveal something about the relative strength of the two positions, which I believe favors 1/3 as an *answer* to the first question. I won't say they favor Elga's *arguments* over Lewis's. That would be reading in too much and let me come back to that. It seems evident that the strength of the Elgan tree over the Lewisian tree is that the former encodes necessity relations on the centered branches that follow from the possible world in which *T* transpired on Sunday night, whereas the latter encodes SB's uncertainties. We have already seen what relying on our uncertainties rather than on what must follow can do in the Ann problem. I suspect the lesson may be repeated here but for better reasons. Lewis keeps *P*(*H*_1_) fixed at 1/2 because he believes that, given no change in relevant information, there should not be a change in subjective probability. Since all agree that *P*(*H*) = 1/2, and since nothing about location is learned upon SB's awakening, there *is* a principled reason for not changing the probabilities. As Lewis notes, he realizes the appeal of Elga's argument, but it is precisely because he finds his own more principled that he sticks to it. There is something to be said about following logic even if it does not lead to intuitive conclusions, and that appears to be what Lewis has done.

While Lewis is correctly principled, both he and Elga mistake what SB's subjective probability on Sunday ought to refer to. Both attribute a subjective probability of 1/2 to SB on Sunday night before she is put to sleep. But what exactly does this probability refer to? Elga and Lewis focus on *P*(*H*), and I believe that is the problem. One should consider what probability SB would assign on Sunday to *H* knowing what she knows about the waking rules of the experiment, and imagining she has just awoken in an asynchronous state in the experiment. Let us call this P^*^(*H*_1_), where the asterisk denotes the counterfactual status of the hypothesis. P^*^(*H*_1_) is the probability of the Stalnaker-type conditional (Stalnaker, 1968) specified in the query, “What is the probability that if you, SB, were to have an asynchronous awakening, then the coin would have come up heads?” We might expand this query, which utilizes a wide-scope probability operator (Over et al., 2013), as follows: “What is the probability that if you, SB, were to have an asynchronous awakening, which in fact you and I know you are not having at the moment, and if you knew all that you know now about the rules of the experiment, then the coin would have come up heads?” In this case, the probability she should assign to P^*^(*H*_1_) equals 1/3, precisely because *P*(*H*) = *P*(*T*) = 1/2, *P*(Monday awakening) = 1, and *P*(Tuesday awakening) = 1/2. Because an asynchronous awakening, *A*, must either be a Monday awakening or a Tuesday awakening, *P*(*A* = Monday) = 2/3. P^*^(*H*_1_) = *P*(*A* = Monday)*P*(*H*) = (2/3)(1/2) = 1/3.

That, on Monday, *P*(*H*_1_) should also equal 1/3 reflects adherence to the dynamic coherence criterion or Bayesian conditioning principle, which states that a probability assessed conditionally on a suppositional event *x* should not differ from the probability assessed conditionally on the actual event *x* (Baratgin and Politzer, 2006). In the Sleeping Beauty problem, *A* may be supposed, contrary to fact, on Sunday night and *A* will be actualized on Monday, and possibly on Tuesday too.

The mislabeling of the event that SB is to consider on Sunday night leads Elga to accept belief revision in the absence of new relevant information. He arrives at a correct answer but forfeits a principle he should have defended. Lewis defends that principle, but ends up with an incorrect estimate because of the initial labeling error. Elgan thirders are therefore right about 1/3 and Lewisian halfers are right to stick to their principles.

Both the Ann and Sleeping Beauty problems illustrate the value of visual representations in reasoning through problems that require people to state their degree of belief in a given proposition. In neither case is the problem's solution clarified by externalizing a natural frequency representation of the problem. Frequency trees and other nested-set-revealing visualizations may facilitate Bayesian reasoning, but so can other forms of visualization, such as (enhanced) logic trees.

The Sleeping Beauty problem also highlights the limits of visualization since nothing in the visualizations offered clarifies the labeling error that I believe lies at the heart of the disagreement; namely, that the proposition being assessed changes from Time 1 (Sunday night) to Time 2 (Monday's asynchronous awakening). Put differently, the visualizations shown in Figure 1 do not represent queries, and it is at the level of query formulation where I believe the controversy first arose. Note too that while the trees in Figure 1 respectively represent Elga's and Lewis's stances on the Sleeping Beauty problem, they do not inherently resolve which stance is more appropriate. At best, they might help other reasoners reach a conclusion by showing in representational terms where disagreement seems to lie.

If my account is correct, it raises the question why P^*^(*H*_1_) could be mistaken for *P*(*H*) by such sharp minds. That it would—namely, that Sunday's apples would be compared with Monday's oranges—is both surprising and a continuing source of my own skepticism in its correctness. Yet, it seems uncontroversial that (a) Elga, Lewis and indeed most commentators on the problem focus their attention on *P*(*H*) when considering SB's Sunday assessment and (b) that this is not well paired with the assessments made upon awakening. To be explicit, the reason it is not well paired is that on Monday, SB must take into account the rules of the experiment, which she perfectly remembers, yet on Sunday she must disregard that knowledge, which is equally at her disposal, in giving her simple credence for heads. Given she is a paragon of rationality, I cannot help but think that she would object to such inconsistency.

### Conflict of interest statement

The author declares that the research was conducted in the absence of any commercial or financial relationships that could be construed as a potential conflict of interest.
